# Smooth Muscle Surprise: Documenting a Primary Ovarian Leiomyosarcoma Case

**DOI:** 10.7759/cureus.69374

**Published:** 2024-09-13

**Authors:** Vallal Kani, Sumithra Arumugam, Karthika Rajendran, Muthuvel Esakki

**Affiliations:** 1 Department of Pathology, Saveetha Medical College and Hospitals, Saveetha Institute of Medical and Technnical Sciences, Saveetha University, Chennai, IND

**Keywords:** leiomyosarcoma, ovarian, primary, smooth muscle, surgery

## Abstract

Primary ovarian leiomyosarcoma (LMS) is an extremely rare and aggressive tumor that originates in the smooth muscle tissue of the ovary. Typically affecting older women, it is diagnosed at an advanced stage and is associated with a poor prognosis, with most patients succumbing within the first year. The effectiveness of adjuvant chemotherapy and radiotherapy remains unclear due to the rarity of reported cases. In this case report, a 66-year-old woman presented with lower abdominal pain persisting for four months, accompanied by weight loss and appetite reduction. Imaging revealed a large, heterogeneous solid lesion in the left adnexa, suggesting a malignant ovarian neoplasm. Surgery was performed, and histopathological analysis confirmed the presence of a high-grade spindle cell sarcoma, consistent with ovarian LMS. Immunohistochemistry showed positive smooth muscle actin (SMA) and negative S100 and Myo D1, supporting the diagnosis. The patient completed two cycles of chemotherapy and was then lost to follow-up. This case highlights the diagnostic challenges and limited treatment options associated with primary ovarian LMS. Given the scarcity of cases, there is no established standard therapy, and the prognosis remains poor. Further research is needed to develop effective therapeutic strategies for this aggressive malignancy.

## Introduction

Primary ovarian leiomyosarcoma (LMS) is an exceptionally rare and highly aggressive cancer, making up less than 1% of ovarian sarcomas and fewer than 3% of all ovarian malignancies [1,2]. LMS originates from the smooth muscle or vascular tissues within the ovary, distinguishing it from other types of ovarian tumors that arise from different cell types [3]. The occurrence of ovarian sarcomas compared to ovarian carcinomas is notably low, with a reported ratio of 1:40. This highlights how rare LMS is within the broader category of ovarian tumors [4]. It primarily affects older women, particularly those over the age of 60. The majority of cases are diagnosed at an advanced stage, which significantly impacts the prognosis. Tragically, most patients with ovarian LMS face a very short survival time, with many succumbing to the disease within the first year after diagnosis [4]. The aggressive nature of ovarian LMS leads to rapid disease progression and frequent metastasis to vital organs such as the lungs and liver. The 5-year survival rate for patients with this condition is alarmingly low, ranging between 20% and 30% [2]. This poor prognosis is partly due to the limited effectiveness of conventional treatments, as the tumor often proves resistant to both chemotherapy and radiotherapy. Surgical resection is currently the primary treatment for ovarian LMS, aimed at removing as much of the tumor as possible. However, due to the extreme rarity of this cancer with fewer than 100 cases reported in the medical literature, there is a lack of consensus on the role of adjuvant therapies such as chemotherapy and radiotherapy [2]. The effectiveness of these treatments remains undetermined, as few studies have been conducted on this specific type of tumor. Consequently, many patients exhibit a minimal response to chemotherapy, which complicates treatment and contributes to the overall poor prognosis [5]. The rarity of ovarian LMS presents significant challenges for establishing standardized treatment protocols. The small number of cases and the lack of comprehensive clinical trials make it difficult to develop evidence-based guidelines. As a result, treatment often relies on individual case management rather than established protocols. This underscores the urgent need for more research to better understand the disease and to develop effective therapeutic strategies.

## Case presentation

A 66-year-old female presented with a history of lower abdominal pain for four months associated with loss of weight and loss of appetite. Her medical history was notable for hypertension, but otherwise, she had no significant past medical or family history. Laboratory studies, including tumor marker assays, were conducted. Serum CA19-9 levels, LDH (lactate dehydrogenase), and CRP (C-reactive protein) levels were elevated, but CA-125 and CEA (carcinoembryonic antigen) levels were within normal limits (Table 1).

**Table 1:** Laboratory values of tumour markers CA 19-9: cancer antigen 19-9, LDH: lactate dehydrogenase, CRP: C-reactive protein, CA 125: cancer antigen 125, CEA: carcinoembryonic antigen.

Parameters	Values	Reference range
CA19-9	142 U/mL	< 37 U/mL
LDH	520 U/L	140-280 U/L
CRP	41.0 mg/L	< 10 mg/L
CA-125	16.4 U/mL	< 35 U/mL
CEA	1.45 ng/mL	<3.00 ng/mL

**Figure 1:**
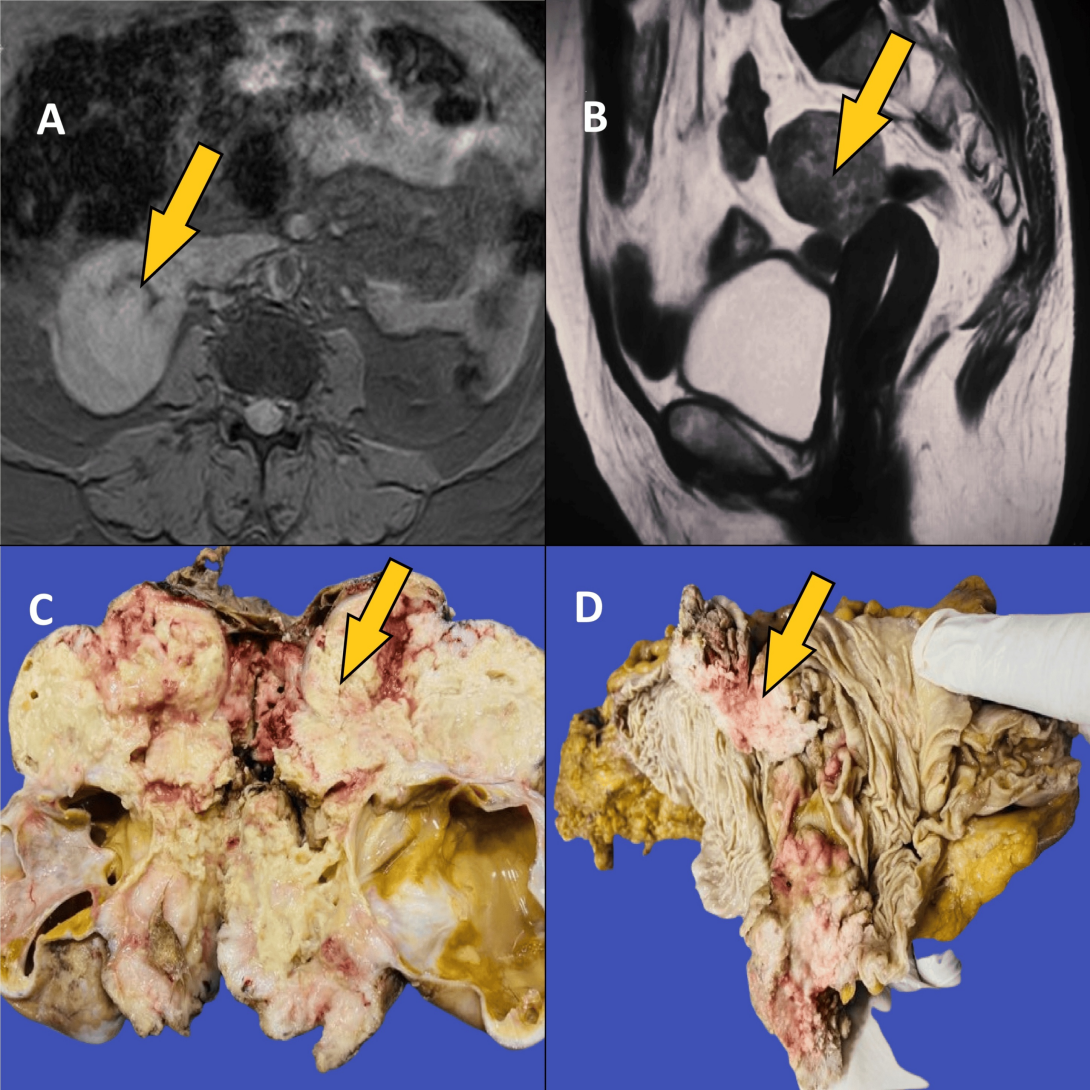
Radiology and gross (A) MRI pelvis showing a large heterogeneous peripherally enhancing, lobulated solid lesion (arrow). (B) Another view of the MRI pelvis shows a large heterogeneous peripherally enhancing, lobulated solid lesion (arrow). (C) Gross specimen showing the cut surface of the ovary with solid and cystic areas of the tumour (arrow). (D) Gross specimen showing the tumour involving the ileum (arrow).

Magnetic resonance imaging (MRI) of the pelvis revealed a large heterogeneous, peripherally enhancing, lobulated solid lesion with central necrosis in the left adnexa, extending into the midline, likely representing a malignant ovarian neoplastic etiology (Figure 1A, B). Exploratory laparotomy with primary cytoreductive surgery (bowel resection with appendectomy and total abdominal hysterectomy) was performed. The specimens were sent for histopathological examination. Macroscopically, a tumor measuring 15 x 12 x 7 cm was noted in the right ovary. The external surface appeared bosselated with an attached friable tissue. The cut surface showed solid and cystic areas (Figure 1C). Solid areas appeared yellowish and friable, while cystic areas contained hairs attached to them. Grossly, the capsule appeared ruptured. The ileum measured 40 cm, and the external surface showed a gray-brown to gray-tan friable mass attached to the mesentery, measuring 8 x 8 x 5 cm, with areas of congestion (Figure 1D).

**Figure 2:**
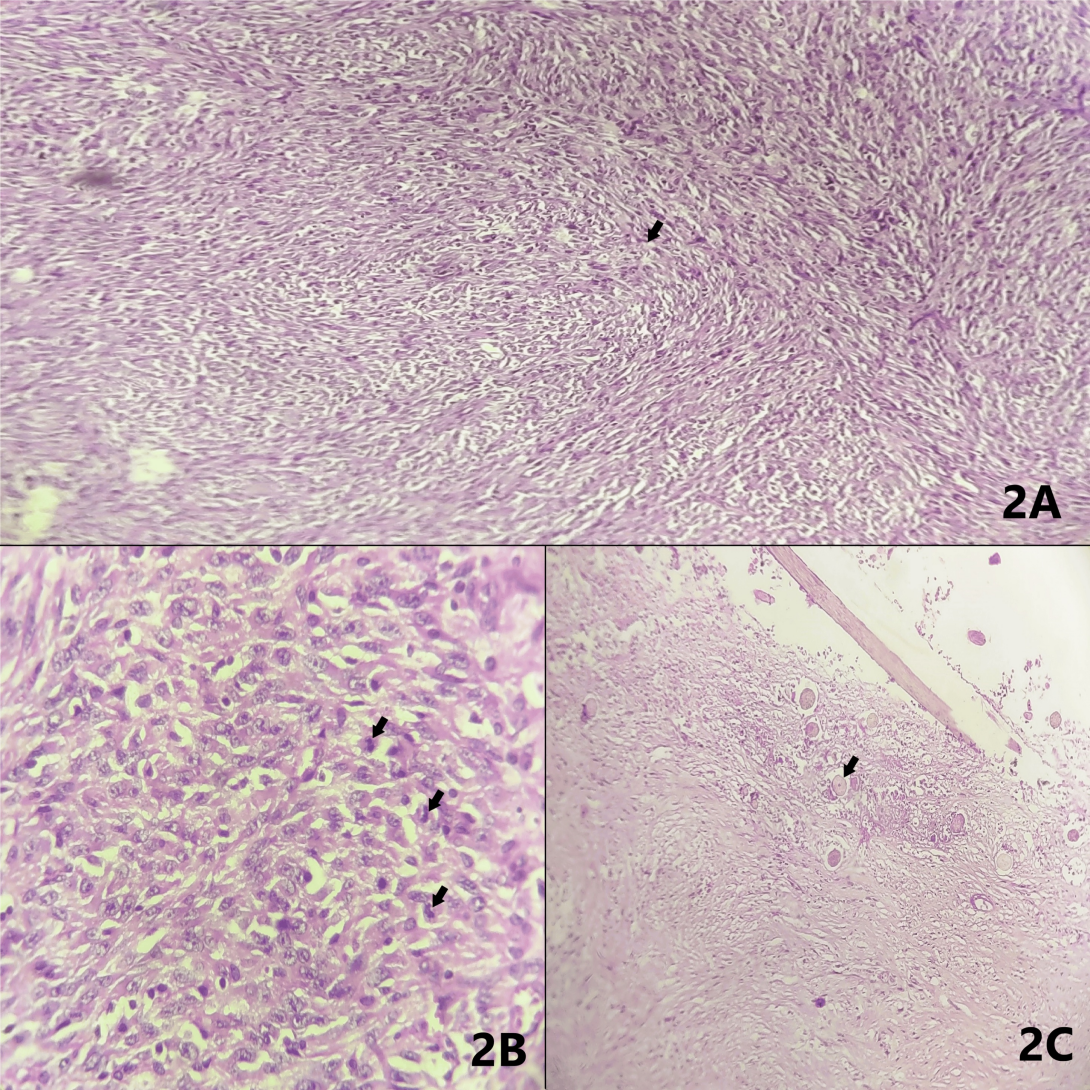
Microscopy (hematoxylin and eosin) (A) Neoplasm composed of fascicles of spindle cells arranged in intersecting bundles having cells that are fusiform and markedly pleomorphic with elongated nuclei, prominent nucleoli and moderately abundant eosinophilic cytoplasm with increased mitotic figures (arrow); magnification: 100X. (B) High-power image showing numerous mitotic figures (arrows); magnification: 400X. (C) Areas showing teratomatous component with hair follicles (arrow); magnification: 100X.

The mucosa appeared unremarkable. The cut surface of the mass was gray-brown, friable, with hemorrhagic areas. The appendix measured 4 cm in length, with unremarkable gross features. The uterus with cervix measured 8 x 5 x 3 cm. The left fallopian tube measured 4 cm in length, and the left ovary measured 3 x 2 x 1 cm. The right tube measured 4 cm in length. The external surface of the uterus was unremarkable. On the cut surface, endometrial thickness was less than 0.1 cm, and myometrial thickness was 1.2 cm. The cut surface of the cervix showed a nabothian cyst measuring 0.5 cm in diameter. In the cut surface of both tubes, the lumen was noted. The external surface of the left ovary showed a cyst measuring 1.5 cm in diameter containing gelatinous material, and on the cut surface, the corpus luteum was seen. Microscopy revealed a histopathological diagnosis based on the CAP (College of American Pathologists) protocol. The right ovarian capsule was ruptured, and the left ovarian capsule was intact, with the bilateral fallopian tubes showing intact serosa. The histological diagnosis was ovarian teratoma with malignant transformation, likely high-grade spindle cell sarcoma (Figures 2A-C). Ovarian surface involvement was present on the right side, but bilateral fallopian tube surface involvement was not identified. The cervix showed chronic non-specific cervicitis with a nabothian cyst. The uterus showed senile cystic atrophic endometrium. The left ovary showed a surface inclusion cyst with corpus albicans. The bilateral tubes and the ileum with the appendix showed no specific pathology.

**Figure 3:**
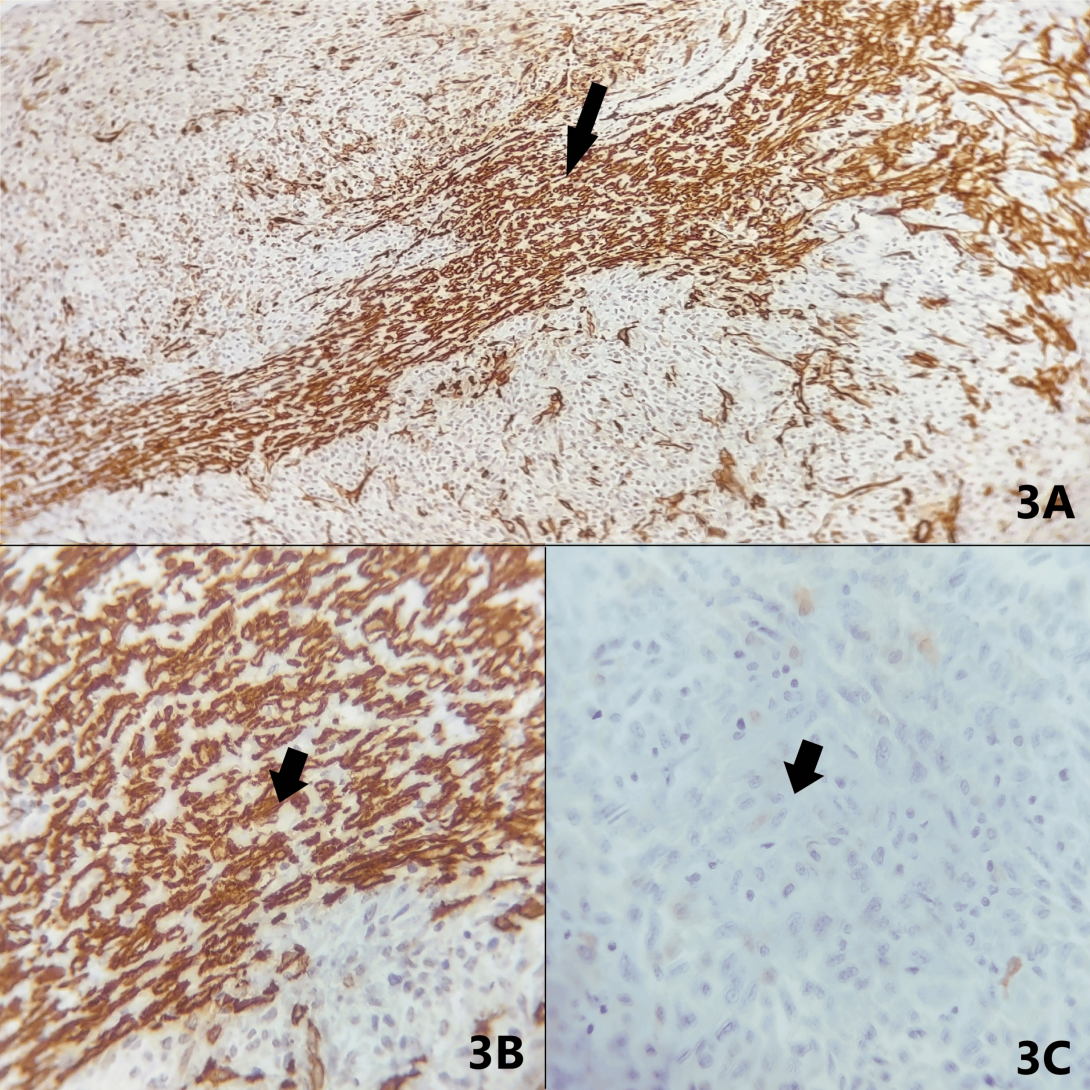
Microscopy-immunohistochemistry (IHC) (A) IHC showing strong cytoplasmic positivity of smooth muscle actin (SMA) in 15-20 % of tumor cells (arrow); magnification: 100X. (B) IHC showing strong cytoplasmic positivity of SMA in 15-20 % of tumor cells (arrow); magnification: 400X. (C) IHC showing S100 negativity in tumor cells (arrow); magnification: 400X.

Immunohistochemistry was performed, and SMA (smooth muscle actin) showed strong cytoplasmic positivity in 15-20% of tumor cells, while S100 was negative in tumor cells, which is suggestive of LMS (Figures 3A-C).

The final impression was given as features suggestive of teratoma with malignant transformation of the right ovary, indicative of LMS. The stage was assigned as pT3c pNx and stage IIIC according to FIGO (International Federation of Gynecology and Obstetrics). The patient was advised to undergo chemotherapy, completed two cycles, and was then lost to follow-up.

## Discussion

Ovarian primary LMS is an exceptionally rare and aggressive malignancy, constituting less than 1% of all ovarian sarcomas [6]. Among ovarian sarcomas, the most common subtypes include rhabdomyosarcoma, endometrial stromal sarcoma, and fibrosarcoma [6]. The genetics and etiology of ovarian sarcomas remain unclear. It is hypothesized that LMS originates from the ovary's smooth muscle components. Although extremely uncommon, uterine leiomyoma migration and malignant transformation of ovarian leiomyoma are also possible [2]. However, LMS remains a significantly understudied entity due to its rarity. The pathogenesis of ovarian sarcomas, including LMS, is still not fully understood, making it challenging to develop targeted therapies or standardized treatment protocols.

The origin of LMS is hypothesized to be linked to the smooth muscle components within the ovary, but other potential sources include mesenchymal totipotent cells, blood vessels, ovarian follicles, ligaments, and the smooth muscles surrounding the corpus luteum [2]. The possibility of malignant transformation of an ovarian leiomyoma or migration of uterine leiomyoma cells to the ovary, although exceedingly rare, has also been suggested [2]. Additionally, some cases of LMS may arise from the smooth muscle differentiation of teratoma cells or migrating smooth muscle cells originating in the uterus [4].

Clinically, LMS often presents with nonspecific symptoms such as abdominal bloating, pelvic pain, and discomfort due to pressure effects on adjacent organs like the bladder and colon. These vague symptoms, as observed in our case, frequently lead to delayed diagnosis and contribute to the advanced stage at which the disease is often detected [4,7]. While LMS predominantly affects postmenopausal women, younger women may also be diagnosed with the disease, typically presenting with large, unilateral ovarian masses [2]. The age range for LMS is broad, with documented cases occurring between 12 and 84 years, although the mean age of onset is around 52.6 years [1].

Histologically, LMS is characterized by fascicles of spindle cells arranged in intersecting bundles. These cells are fusiform and pleomorphic, with elongated nuclei, prominent nucleoli, and eosinophilic cytoplasm. The nuclei have blunted or truncated ends and dense cytoplasm [2,7]. The presence of hypercellularity, nuclear atypia, pleomorphism, coagulative necrosis, and a high mitotic rate (greater than 5 mitoses per 10 high-power fields) are key diagnostic criteria for LMS [1]. These features are critical in distinguishing LMS from other spindle-cell neoplasms, such as leiomyoma, fibrothecoma, fibrosarcoma, endometrial stromal sarcoma, spindle cell carcinoma, and gastrointestinal stromal tumor (GIST) [7].

Immunohistochemical analysis is a valuable tool in diagnosing LMS. Typically, LMS cells exhibit strong positivity for smooth muscle markers such as SMA (smooth muscle actin), Desmin, Vimentin, and Caldesmon, while being negative for markers like S100 and Cytokeratins [2]. In the present case, the tumor demonstrated strong cytoplasmic positivity for SMA and negativity for S100, consistent with a diagnosis of LMS.

Due to its aggressive nature, LMS is usually diagnosed at an advanced stage, with tumors often exceeding 10 cm in size [4]. The prognosis for LMS is generally poor, with most patients experiencing rapid disease progression and high mortality rates within the first year of diagnosis [4]. In our case, the patient was lost to follow-up, underscoring the challenges in managing this disease and the importance of long-term monitoring.

The treatment of LMS typically involves surgery, with postoperative radiotherapy used to control local disease and chemotherapy employed to prevent distant metastasis [7]. However, the efficacy of adjuvant therapies remains uncertain, given the limited number of cases reported in the literature. Adjuvant therapy may be more beneficial in cases where the tumor is operable but carries a poor prognosis due to factors like large size or advanced stage at diagnosis [1].

Given the rarity of LMS, with fewer than 100 cases documented globally, there is a critical need for more extensive research to better understand its etiology, behavior, and optimal treatment strategies. Collaborative efforts across multiple institutions and the inclusion of LMS cases in larger oncological studies may provide more definitive insights and help develop evidence-based guidelines for managing this rare and challenging malignancy.

## Conclusions

Primary ovarian LMS is an exceedingly rare and aggressive malignancy with a poor prognosis due to its advanced presentation and limited treatment options. The rarity of this tumor presents significant challenges in diagnosis, treatment, and predicting outcomes. In this case, the patient presented with nonspecific symptoms, and the diagnosis was confirmed post-surgically through histopathological and immunohistochemical analysis. Despite surgical intervention and chemotherapy, the prognosis remains uncertain, emphasizing the need for continued follow-up. This case underscores the importance of considering primary ovarian LMS as a differential diagnosis, particularly in postmenopausal women presenting with large adnexal masses. Given the limited number of documented cases, further research is necessary to establish standardized treatment protocols and improve the survival rate for patients diagnosed with this rare and aggressive tumor.
